# Jottings

**Published:** 1909-10-09

**Authors:** 


### JOTTINGS.

The Lord Mayor recently laid the foundation-stone of a home for the nurses to be built in the grounds of the City of London Asylum at Stone, near Dartford.

The foundation-stone of a new children's ward at Royal Boscombe and West Hants Hospital was laid last Monday. The cost of the work is £4,000.

The third annual demonstration in aid of the North Lonsdale Hospital, Barrow, was held on September 18. The whole town was *en fête* throughout the day, and eagerly supported the demonstration, for which it had taken weeks and months to prepare.

